# Surveillance Systems to Track Progress Toward Global Polio Eradication — Worldwide, 2012–2013

**Published:** 2014-04-25

**Authors:** Alexandra Levitt, Ousmane M. Diop, Rudolf H. Tangermann, Fem Paladin, Jean Baptiste Kamgang, Cara C. Burns, Paul J. Chenoweth, Ajay Goel, Steven G.F. Wassilak

**Affiliations:** 1 Office of Infectious Diseases, CDC; 2 Polio Eradication Department, World Health Organization, Geneva, Switzerland; 3 Global Immunization Division, Center for Global Health, CDC; 4 Division of Viral Diseases, National Center for Immunization and Respiratory Diseases, CDC

In 2012, the World Health Assembly of the World Health Organization (WHO) declared completion of polio eradication a programmatic emergency (1). Polio cases are detected through surveillance of acute flaccid paralysis (AFP) cases and subsequent testing of stool specimens for polioviruses (PVs) at WHO-accredited laboratories within the Global Polio Laboratory Network (GPLN). AFP surveillance is supplemented by environmental surveillance, testing sewage samples from selected sites for PVs. Virologic surveillance, including genomic sequencing to identify isolates by genotype and measure divergence between isolates, guides Global Polio Eradication Initiative (GPEI) activities by confirming the presence of PV, tracking chains of PV transmission, and highlighting gaps in AFP surveillance quality. This report provides AFP surveillance quality indicators at national and subnational levels during 2012–2013 for countries that experienced PV cases during 2009–2013 in the WHO African Region (AFR) and Eastern Mediterranean Region (EMR), the remaining polio-endemic regions (2). It also summarizes the results of environmental surveillance and reviews indicators assessing the timeliness of reporting of PV isolation and of virus strain characterization globally. Regional-level performance indicators for timely reporting of PV isolation were met in five of six WHO regions in 2012 and 2013. Of 30 AFR and EMR countries that experienced cases of PV (wild poliovirus [WPV], circulating vaccine-derived poliovirus [cVDPV], or both) during 2009–2013, national performance indicator targets for AFP surveillance and collection of adequate specimens were met in 27 (90%) countries in 2012 and 22 (73%) in 2013. In 17 (57%) countries, ≥80% of the population lived in subnational areas meeting both AFP performance indicators in 2012, decreasing to 13 (43%) in 2013. To achieve polio eradication and certify interruption of PV transmission, intensive efforts to strengthen and maintain AFP surveillance are needed at subnational levels, including in field investigation and prompt collection of specimens, particularly in countries with current or recent active PV transmission.

## AFP Surveillance

Paralysis, the long-lasting manifestation of clinical poliomyelitis, is a rare outcome of WPV and cVDPV infections (<1%). AFP surveillance detects recent acute paralytic illness of any cause, including poliomyelitis caused by WPV or VDPV. Standardized GPEI performance indicators are used to evaluate the quality of AFP surveillance and changes over time and to identify surveillance gaps where PV transmission might go undetected. The indicator used to determine if surveillance is sufficiently sensitive to detect PV circulation is the annual proportion of AFP cases that are negative for WPV and VDPV (nonpolio AFP [NPAFP]) among children aged <15 years. Countries in WHO regions certified as polio-free* should achieve an annual NPAFP rate of ≥1 case per 100,000 population aged <15 years; all other countries† should achieve annual rates of ≥2 cases per 100,000. To ensure sufficiently complete and reliable laboratory analysis, ≥80% of AFP cases should have two stool specimens collected within 14 days of paralysis onset, ≥24 hours apart, arriving in good condition at an accredited GPLN laboratory (“adequate” specimens). Because national data can mask heterogeneous subnational performance, AFP surveillance quality indicators are applied to subnational areas, and the proportion of the national population residing in subnational areas where both indicator targets are met was assessed (Table 1, Figure).

In 2012, AFP surveillance detected WPV transmission in five countries, including three countries with uninterrupted, endemic WPV transmission (Afghanistan, Nigeria, and Pakistan), one previously polio-free country with reestablished WPV transmission (Chad), and one polio-free country with an outbreak after importation (Niger) (Table 1). In 2013, WPV cases were detected in eight countries, including the three countries with endemic WPV (Afghanistan, Nigeria, and Pakistan), and five countries affected by outbreaks after importation (Cameroon, Ethiopia, Kenya, Somalia, and Syria). All WPV cases were type 1 (WPV1).

cVDPV-associated polio cases were detected in eight AFR and EMR countries in 2012 (Afghanistan, Chad, Democratic Republic of the Congo [DRC], Kenya, Nigeria, Pakistan, Somalia, and Yemen) and in eight countries in 2013 (Afghanistan, Cameroon, Chad, Niger, Nigeria, Pakistan, Somalia, and Yemen) (Table 1) (3). All cVDPVs isolated from persons with AFP during 2012 and 2013 were type 2, except for those in Yemen (type 3).

Of 25 AFR countries with PV transmission during 2009–2013, the NPAFP national target was met in all countries in 2012, and all except Gabon in 2013. The national target for adequate specimen collection was met in all countries except Cameroon and Gabon in 2012, and in all except Cameroon, Ethiopia, Gabon, Guinea, Niger, Republic of the Congo, and Senegal in 2013. Twelve of the 25 countries had all subnational areas meeting the subnational NPAFP rate target in 2012 or in 2013. Only three of the 25 countries had all subnational areas reporting adequate specimen collection in 2013, compared with 10 in 2012. (Table 1, Figure). In only nine (36%) countries, ≥80% of the population lived in areas meeting both subnational indicators during both 2012 and 2013 (Benin, Côte d’Ivoire, Liberia, Mali, Mauritania, Mozambique, Nigeria, South Sudan, and Togo).

Of five EMR countries that experienced PV transmission during 2009–2013, only Syria in 2012 and 2013 failed to meet the national target for NPAFP rate, and only Syria failed to meet the national standard for specimen adequacy in 2013. However, only two of the five had all subnational areas meeting the target for NPAFP rate in 2012 and three of the five in 2013; one country in each year had all subnational areas reporting adequate specimen collection. Nonetheless, the target of having ≥80% of the population living in areas meeting both subnational indicators was reached in all polio-affected countries in EMR during 2012–2013, except Somalia and Syria in 2012 and Syria in 2013.

## Environmental Surveillance

The sampling and testing of sewage complements AFP surveillance by identifying PV transmission that might occur in the absence of detected AFP cases (2). Environmental surveillance has been established in WPV-endemic countries (Afghanistan since September 2013, Nigeria since 2011, and Pakistan since 2009) and in countries without active WPV transmission currently (India, Egypt, and 19 countries in the WHO European Region). Active WPV1 transmission without detection of polio cases was identified in Israel, the West Bank, and Gaza in 2013 (4,5). Genomic sequencing and phylogenetic analyses indicate that the WPV1 originated in Pakistan and is closely linked to WPV1 isolated from two sewage specimens collected in December 2012 in Cairo, Egypt (2) and to WPV1 cases detected in 2013 in Syria (6,7), indicating widespread circulation in the Middle East during the end of 2012 and early 2013. In Afghanistan, no WPV or VDPV have been detected in the few samples collected in Kandahar city and tested since September 2013.

In Nigeria, sampling is currently conducted at 29 sites in seven states and the Federal Capital Territory. In 2012, WPV1 was isolated from two sewage samples from Kano state, and from multiple samples from Sokoto state when WPV-confirmed AFP cases were widely reported in both states. In 2013, WPV1 was isolated from one sewage sample in Kano (February), from three samples collected in Sokoto (March–April), and one sample collected from a new site in Borno state (October). Continued VDPV2 circulation and transmission of cVDPV2 imported from Chad was documented during 2012–2013 through VPDV2 isolation from samples collected in Sokoto (continued circulation), and in Kano and Borno (Chad-related). No WPV or VDPV has been detected at environmental surveillance sites established in 2013 in other Nigerian states.

What is already known on this topic?

Polio cases are detected through surveillance of acute flaccid paralysis (AFP) cases with stool specimens tested for polioviruses (PVs) at accredited laboratories within the Global Polio Laboratory Network. Some countries also test for polioviruses in samples taken from sewage. Genomic sequence analysis allows the Global Polio Eradication initiative to monitor pathways of PV transmission, both of wild poliovirus (WPV) and circulating vaccine-derived poliovirus (cVDPV).

What is added by this report?

Of 30 countries in the World Health Organization African and Eastern Mediterranean regions with transmission of WPV or cVDPV during 2009–2013, those meeting national performance indicator targets for AFP surveillance and collection of adequate specimens declined from 27 (90%) in 2012 to 22 (73%) in 2013, primarily because of surveillance weaknesses in the African Region. The number of subnational areas meeting both AFP performance indicators in 2012 and 2013 also declined in many countries of the African Region. Environmental surveillance often found evidence of PV circulation in the absence of detected AFP cases.

What are the implications for public health practice?

WPV outbreaks in previously polio-free countries in Africa and the Middle East are reminders that all countries remain at risk as long as WPV continues to circulate in any one country. Intensive efforts are needed to strengthen and maintain AFP surveillance, including analysis of the reasons for surveillance weaknesses, training of surveillance staff, and enhanced supervision in field investigation and collection of specimens, in countries with current or recent active poliovirus transmission.

In Pakistan, sampling is currently conducted at 27 sites in four provinces. The overall proportion of sewage samples positive for WPV1 decreased from 67% in 2011 to 20% in 2013. Environmental surveillance detected continuous WPV1 circulation in Hyderabad (in southern Sindh Province) into mid-2013, without any corresponding WPV1-confirmed AFP case reported in the same area for >12 months. During 2013, WPV1 was isolated sporadically from samples collected in Quetta, Karachi, and from sites in Punjab Province.

## Global Polio Laboratory Network

The GPLN consists of 146 WHO-accredited PV laboratories in all WHO regions. GPLN member laboratories follow standardized protocols to 1) isolate and identify PVs, 2) differentiate the three PV serotypes, and 3) characterize PVs as WPV, Sabin-like PV, and VDPV by intratypic differentiation [ITD]) (8) and genomic sequencing. Results of sequencing are also used to monitor pathways of PV transmission by comparing the nucleotide sequence of the VP1 region of the genomes from PV isolates. The two standard laboratory timeliness indicators for stool specimen processing are that laboratories should report ≥80% of PV isolation results within 14 days of receiving samples and ≥80% of ITD results within 7 days of receipt of isolates. The programmatic indicator standard combining field and laboratory performance is to report ITD results for ≥80% of isolates within 60 days of paralysis onset of AFP cases. This indicator takes into account the entire interval from onset of paralysis through case notification, investigation, and specimen collection, transport, and testing (EMR uses a 45-day timeframe). In addition to timeliness, the accuracy and quality of testing at GPLN member laboratories is monitored through an annual accreditation program of onsite reviews and proficiency testing for viral isolation, ITD, and sequencing procedures.

During 2012–2013, GPLN laboratories met timeliness indicators for PV isolation in five of six WHO regions in each year (Table 2) and reporting indicators for receipt to ITD results in five of six regions in 2012 and all regions in 2013. The overall timeless indicator for onset to ITD results was met in all regions in both years. The GPLN tested a total of 215,629 stool specimens collected from persons with AFP in 2012 and 197,658 in 2013. In 2013, an additional 10,871 stool specimens from contacts of AFP cases, 3,223 stool specimens from other investigations, and 2,537 environmental samples were tested. In 2012, 395 WPV isolates were detected from AFP samples compared with 723 detected in 2013 (an 83% increase). In addition, 125 VDPV isolates were detected from AFP cases in 2012, compared with 65 VDPV isolates detected in 2013 (a 52% decrease).

During 2012, genomic sequencing identified two WPV1 genotypes and one WPV type 3 genotype in samples from AFR countries: type 1 West Africa-B1 (WEAF-B1) genotype was detected in Nigeria, Niger, and Chad; type 1 WEAF-B2 type 1 genotype and type 3 WEAF-B genotype were detected only in Nigeria. In the WHO Eastern Mediterranean Region, type 1 South Asia (SOAS) and type 3 SOAS genotypes were detected in 2012. In 2013, only type 1 WEAF-B1 and SOAS genotypes were isolated. When genomic sequencing of an isolate detects ≥1.5% nucleotide divergence in the VP1-coding region from previously identified PV isolates, this highlights prolonged undetected circulation and quality gaps in field AFP surveillance, even though it is not always obvious to determine where transmission was missed. Sequence analysis indicated that WPV cases were likely being missed by AFP surveillance during 2012–2013 in Afghanistan, Cameroon, Chad, Niger, Nigeria, Pakistan, and Syria; cVDPV cases during 2012–2013 were also likely missed in Afghanistan, Nigeria, and Somalia.

### Discussion

During 2012–2013, 12 AFR and EMR countries were affected by WPV or cVDPV cases. National and subnational AFP performance indicators highlighted weak performance in seven of these 12 countries. Virologic analysis and environmental surveillance indicated weaknesses in three of the other countries even when AFP indicators were met. These surveillance weaknesses have limited the ability to rapidly detect WPV introductions and better target GPEI immunization activities in areas with transmission.

AFP surveillance indicators remained strong or improved during 2012–2013 in some African countries where WPV or cVDPV outbreaks occurred in the past, including Benin, Côte d’Ivoire, Liberia, Mali, Mauritania, Mozambique, Nigeria, Republic of the Congo, Sierra Leone, South Sudan, and Togo. However, indicators showing surveillance weaknesses were reported in countries with recent circulation within or near the country, including Cameroon, Central African Republic, Ethiopia, Gabon, Kenya, and Niger (as well as Guinea and Senegal), where deficiencies were primarily related to relatively low proportions of AFP cases with adequate specimens. The outbreaks in the Horn of Africa and Central Africa potentially could have been controlled more promptly if they had been detected earlier by surveillance meeting all performance standards; the risk remains for delayed detection of spread to some neighboring countries. The proportion of adequate specimens can be increased in AFP surveillance by careful review of the reasons for late detection or investigation, refresher training of surveillance and investigative staff, and enhanced field supervision. AFP surveillance indicators were generally strong in polio-affected countries in EMR, with the exception of Syria, where surveillance efforts are limited by civil conflict and displacement of populations.

The occurrence of WPV outbreaks in previously polio-free countries in Africa and the Middle East is a reminder that all countries remain at risk as long as WPV continues to circulate in any one country. For prompt detection of WPV introduction and for ultimate certification of polio eradication, polio-free countries should maintain certification-standard surveillance performance indicators. The *GPEI Polio Eradication and Endgame Strategic Plan for 2013–2018* (9) prioritizes efforts to maintain and improve PV surveillance at all administrative levels throughout each country, including active AFP surveillance at health facilities, with special attention to populations with a high risk for undetected PV transmission (e.g., mobile and displaced populations). In countries with large populations (e.g., DRC, Nigeria, and Pakistan), surveillance performance needs to be monitored closely at lower administrative levels (e.g., districts, rather than at states/provinces). Environmental surveillance continues to augment AFP surveillance and will be expanded within selected high-risk countries and those in which WPV is endemic. Intensive efforts to strengthen and maintain AFP surveillance are needed in all countries with current or recent active PV transmission to better target GPEI immunization activities in 2014.

## Figures and Tables

**FIGURE:**
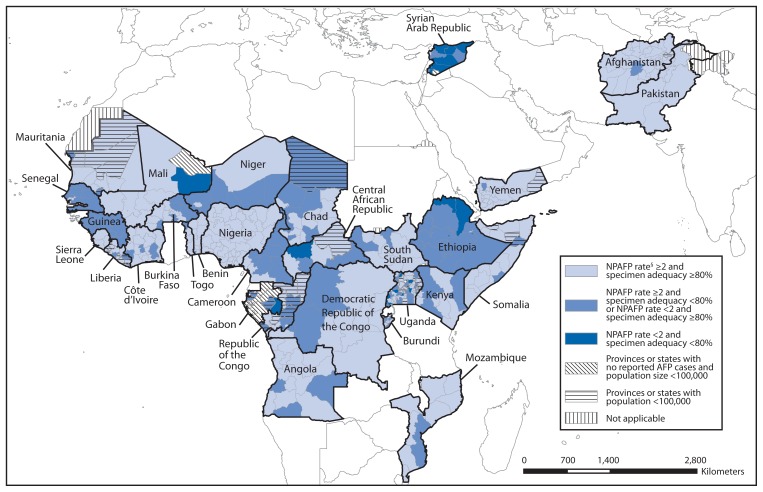
Combined performance indicators for the quality of acute flaccid paralysis (AFP) surveillance^*^ in subnational areas (states and provinces) of 30 countries that were polio-affected during 2009–2013 — World Health Organization African and Eastern Mediterranean regions, 2013^†^ **Abbreviation:** NPAFP = nonpolio AFP. ^*^ The Global Polio Eradication Initiative has set the following targets for countries with current or recent wild poliovirus transmission and their states/provinces: 1) NPAFP detection rate of two or more cases per 100,000 persons aged <15 years, and 2) adequate stool specimen collection from ≥80% of AFP cases, with specimen adequacy defined as two specimens collected ≥24 hours apart, both within 14 days of paralysis onset, shipped on ice or frozen packs, and arriving in good condition (without leakage or desiccation) at a World Health Organization–accredited laboratory. ^†^ Data are for AFP cases with onset during 2013, reported as of March 31, 2014. ^§^ Per 100,000 persons aged >15 years.

**TABLE 1:** National and subnational acute flaccid paralysis (AFP) surveillance indicators and number of confirmed wild poliovirus (WPV) and circulating vaccine-derived poliovirus (cVDPV) cases, among countries with poliovirus transmission during 2009–2013 within the African and Eastern Mediterranean regions of the World Health Organization (WHO) and regional indicators, 2012 and 2013.*

WHO region/Country†	2012								2013							
	
	AFP cases	Regional/ National NPAFP rate§	Subnational areas with NPAFP rate ≥2 (%)	Regional/ National AFP cases with adequate specimens¶ (%)	Subnational areas with ≥80% adequate specimens (%)	Population in areas meeting both subnational indicators** (%)	Confirmed WPV cases††	Confirmed cVDPV cases§§	AFP cases	Regional/ National NPAFP rate§	Subnational areas with NPAFP rate ≥2 (%)	Regional/ National AFP cases with adequate specimens¶ (%)	Subnational areas with ≥80% adequate specimens (%)	Population in areas meeting both subnational indicators** (%)	Confirmed WPV cases††	Confirmed cVDPV cases§§
**African**	18,075	4.8	—	(90)	—	—	128	40	20,264	5.3	—	(91)	—	—	80	13
Angola	319	3.1	(94)	(92)	(100)	(98)	—	—	310	2.9	(89)	(92)	(94)	(79)	—	—
Benin	153	3.6	(92)	(92)	(92)	(87)	—	—	180	4.3	(100)	(91)	(92)	(95)	—	—
Burkina Faso	321	4.0	(93)	(89)	(100)	(100)	—	—	292	3.7	(86)	(85)	(71)	(65)	—	—
Burundi	117	2.9	(71)	(98)	(100)	(72)	—	—	96	2.4	(59)	(91)	(88)	(49)	—	—
Cameroon	336	2.8	(100)	(79)	(60)	(56)	—	—	483	4.3	(100)	(77)	(30)	(25)	4	4
CAR	124	6.0	(100)	(85)	(86)	(88)	—	—	60	2.6	(57)	(90)	(71)	(36)	—	
Chad	418	6.7	(100)	(82)	(67)	(67)	5	12	500	8.6	(100)	(82)	(56)	(56)	—	4
Côte d’Ivoire	406	4.3	(100)	(83)	(83)	(85)	—	—	455	4.9	(100)	(88)	(83)	(87)	—	—
DRC	1,867	4.4	(100)	(86)	(91)	(86)	—	17	2,011	4.8	(100)	(83)	(73)	(70)	—	—
Ethiopia	1,156	2.8	(91)	(85)	(55)	(69)	—	—	1,164	2.8	(64)	(71)	(9)	(0)	9	—
Gabon	25	2.5	(63)	(76)	(75)	(16)	—	—	6	0.6	(67)	(17)	(0)	(0)	—	—
Guinea	187	3.3	(100)	(97)	(100)	(100)	—	—	224	4.0	(100)	(54)	(0)	(0)	—	—
Kenya	714	4.2	(100)	(92)	(100)	(100)	—	3	637	3.5	(88)	(84)	(88)	(65)	14	—
Liberia	56	3.2	(73)	(100)	(100)	(70)	—	—	50	2.9	(80)	(98)	(100)	(86)	—	—
Mali	266	3.4	(75)	(94)	(88)	(92)	—	—	243	3.1	(88)	(88)	(88)	(96)	—	—
Mauritania	78	5.7	(100)	(95)	(100)	(100)	—	—	58	4.2	(100)	(93)	(85)	(90)	—	—
Mozambique	320	3.1	(100)	(89)	(100)	(100)	—	—	333	3.3	(100)	(89)	(80)	(85)	—	—
Niger	368	4.3	(88)	(80)	(50)	(55)	1	—	338	4.1	(100)	(75)	(25)	(8)	—	1
Nigeria§§	7,239	8.7	(100)	(95)	(97)	(96)	122	8	8,641	10.5	(100)	(96)	(100)	(100)	53	4
Republic of the Congo	58	2.7	(64)	(84)	(64)	(19)	—	—	106	5.2	(100)	(79)	(64)	(78)	—	—
Senegal	160	2.7	(73)	(81)	(55)	(50)	—	—	231	3.7	(91)	(68)	(18)	(7)	—	—
Sierra Leone	168	6.3	(75)	(95)	(100)	(79)	—	—	171	6.4	(60)	(92)	(100)	(79)	—	—
South Sudan	325	4.3	(100)	(95)	(90)	(97)	—	—	295	3.8	(90)	(94)	(90)	(87)	—	—
Togo	94	2.9	(100)	(97)	(100)	(100)	—	—	155	4.7	(100)	(85)	(83)	(87)	—	—
Uganda	472	3.2	(65)	(88)	(71)	(52)	—	—	486	3.3	(71)	(87)	(77)	(51)	—	—
**Eastern Mediterranean**	11,119	5.2	—	(91)	—	—	95	28	11,520	5.2	—	(90)	—	—	327	50
Afghanistan	1,829	10.2	(100)	(92)	(94)	(91)	37	9	1,897	10.8	(100)	(94)	(97)	(97)	14	3
Pakistan	5,036	5.6	(88)	(89)	(88)	(98)	58	16	4,778	5.2	(88)	(90)	(100)	(99)	93	45
Somalia	148	2.8	(79)	(98)	(100)	(56)	—	1	546	6.4	(100)	(88)	(89)	(93)	194	1
Syria	109	1.1	(15)	(85)	(62)	(10)	—	—	171	1.3	(15)	(64)	(38)	(4)	25	—
Yemen	474	4.0	(100)	(93)	(95)	(98)	—	2	614	5.2	(100)	(92)	(91)	(84)	—	1

**Abbreviations:** NPAFP = nonpolio AFP; DRC = Democratic Republic of the Congo; CAR = Central African Republic.

* Data as of March 25, 2014.

† Regional NPAFP rates use United Nations Development Programme populations as denominators, and therefore tend to be higher than country rates, which use their summed subnational populations as denominators. Regional data available at http://apps.who.int/immunization_monitoring/en/diseases/poliomyelitis/case_count.cfm.

§ Per 100,000 persons aged <15 years.

¶ Standard WHO target is adequate stool specimen collection from ≥80% of AFP cases, in which two specimens are collected within 14 days of paralysis onset ≥24 hours apart, shipped on ice or frozen ice packs and arriving in good condition in a WHO-accredited laboratory. Stool specimen adequacy proportions from regions do not include the criteria of good specimen condition or time between specimens.

** For all subnational areas regardless of the population size.

†† Data at WHO as of April 1, 2014.

§§ Data at WHO as of April 1, 2014; cVDPV are VDPV associated with two or more cases of AFP.

**TABLE 2:** Number of poliovirus (PV) isolates from stool specimens of persons with acute flaccid paralysis and timing of results, by World Health Organization (WHO) region, 2012 and 2013*

WHO region	2012							2013						
	
	No. of specimens	No. of PV isolates			PV isolation results on time¶ (%)	ITD results within 7 days** (%)	ITD results within 60 days†† (%)	No. of specimens	No. of PV isolates			PV isolation results on time¶ (%)	ITD results within 7 days** (%)	ITD results within 60 days†† (%)
	
		Wild	Sabin†	cVDPV§					Wild	Sabin†	cVDPV§			
African	39,710	221	2,629	43	(95)	(99)	(93)	42,316	598	2,861	12	(92)	(88)	(84)
Americas	1,926	0	31	0	(77)	(91)	(100)	1,672	0	33	0	(80)	(95)	(91)
Eastern Mediterranean	26,626	174	930	71	(94)	(99)	(99)	20,783	125	626	53	(99)	(98)	(97)
European	3,167	0	66	2	(96)	(75)	(88)	3,404	0	37	0	(99)	(93)	(86)
South-East Asia	129,106	0	3,470	1	(98)	(87)	(100)	116,179	0	3,274	0	(98)	(91)	(98)
Western Pacific	15,094	0	223	8	(98)	(93)	(84)	13,304	0	241	0	(65)	(100)	(99)
Total	215,629	395	7,349	125	(93)	(91)	(94)	197,658	723	7,072	65	(89)	(94)	(93)

**Abbreviations:** cVDPV = circulating vaccine-derived poliovirus; ITD = intratypic differentiation.

* Data as of April 1, 2014.

† Either concordant Sabin-like results in ITD test and VDPV screening, or <1% sequence difference compared with Sabin vaccine virus (<0.6% for type 2).

§ For PV types 1 and 3, 10 or more VP1 nucleotide differences from the respective PV; for PV type 2, six or more VP1 nucleotide differences from Sabin type 2 PV.

¶ Results reported within 14 days for laboratories in the following WHO regions: African, Americas, Eastern Mediterranean, South-East Asia, and Western Pacific

(28 days for China in 2012; change in procedures in China implemented during 2013 and 14 day criterion applied in 2013). Results reported within 28 days for the European Region.

** Results of ITD reported within 7 days of receipt of specimen.

†† Results reported within 60 days of paralysis onset for all WHO regions except Eastern Mediterranean Region, which reported within 45 days of paralysis onset.

## Footnotes

* American, European, South-East Asia, and Western Pacific regions. Data for countries of these regions and regional values are available at http://www.who.int/vaccines/immunization_monitoring/en/diseases/poliomyelitis/case_count.cfm.

† Countries in the African and Eastern Mediterranean regions. Although the South-East Asia Region has been recently certified polio-free, countries are still expected to reach the two or more NPAFP rate standard.
